# The Glycolytic Metabolite, Fructose-1,6-bisphosphate, Blocks Epileptiform Bursts by Attenuating Voltage-Activated Calcium Currents in Hippocampal Slices

**DOI:** 10.3389/fncel.2018.00168

**Published:** 2018-06-15

**Authors:** Li-Rong Shao, Guangxin Wang, Carl E. Stafstrom

**Affiliations:** ^1^Division of Pediatric Neurology, Department of Neurology, School of Medicine, Johns Hopkins University, Baltimore, MD, United States; ^2^Department of Medicine, Qilu Children’s Hospital, Shandong University, Jinan, China

**Keywords:** seizure, metabolism, Ca^2+^ current, neural excitability, patch-clamp, glycolysis, the pentose-phosphate pathway

## Abstract

Manipulation of metabolic pathways (e.g., ketogenic diet (KD), glycolytic inhibition) alters neural excitability and represents a novel strategy for treatment of drug-refractory seizures. We have previously shown that inhibition of glycolysis suppresses epileptiform activity in hippocampal slices. In the present study, we aimed to examine the role of a “branching” metabolic pathway stemming off glycolysis (i.e., the pentose-phosphate pathway, PPP) in regulating seizure activity, by using a potent PPP stimulator and glycolytic intermediate, fructose-1,6-bisphosphate (F1,6BP). Employing electrophysiological approaches, we investigated the action of F1,6BP on epileptiform population bursts, intrinsic neuronal firing, glutamatergic and GABAergic synaptic transmission and voltage-activated calcium currents (I_Ca_) in the CA3 area of hippocampal slices. Bath application of F1,6BP (2.5–5 mM) blocked epileptiform population bursts induced in Mg^2+^-free medium containing 4-aminopyridine, in ~2/3 of the slices. The blockade occurred relatively rapidly (~4 min), suggesting an extracellular mechanism. However, F1,6BP did not block spontaneous intrinsic firing of the CA3 neurons (when synaptic transmission was eliminated with DNQX, AP-5 and SR95531), nor did it significantly reduce AMPA or NMDA receptor-mediated excitatory postsynaptic currents (EPSC_AMPA_ and EPSC_NMDA_). In contrast, F1,6BP caused moderate reduction (~50%) in GABA_A_ receptor-mediated current, suggesting it affects excitatory and inhibitory synapses differently. Finally and unexpectedly, F1,6BP consistently attenuated I_Ca_ by ~40% without altering channel activation or inactivation kinetics, which may explain its anticonvulsant action, at least in this *in vitro* seizure model. Consistent with these results, epileptiform population bursts in CA3 were readily blocked by the nonspecific Ca^2+^ channel blocker, CdCl_2_ (20 μM), suggesting that these bursts are calcium dependent. Altogether, these data demonstrate that the glycolytic metabolite, F1,6BP, blocks epileptiform activity via a previously unrecognized extracellular effect on I_Ca_, which provides new insight into the metabolic control of neural excitability.

## Introduction

Accumulating evidence suggests that neuronal activity is tightly coupled to energy metabolism (Magistretti and Pellerin, 1996; Ames, 2000), which is not surprising given that the brain consumes ~20% of the body’s energy. Metabolic manipulation provides a unique strategy to regulate excessive neuronal activity (such as seizures), which is particularly important given that at least 1/3 of patients with epilepsy are resistant to drug treatment. One relevant example of seizure control through metabolism is the low-carbohydrate, adequate-protein, high-fat ketogenic diet (KD), which has proven to be highly effective in controlling many types of drug-refractory seizures (Freeman and Vining, 1998; Stafstrom and Rho, 2012; Thakur et al., 2014). More recently, a new approach has been to alter energy metabolism by inhibiting glycolysis with a non-metabolizable glucose analog, 2-deoxy-glucose (2-DG). Partial inhibition of glycolysis with 2-DG blocks seizure activity in brain slices and in several animal models of seizures and epileptogenesis (Garriga-Canut et al., 2006; Stafstrom et al., 2009; Forte et al., 2016; Shao and Stafstrom, 2017).

**Figure 1:**
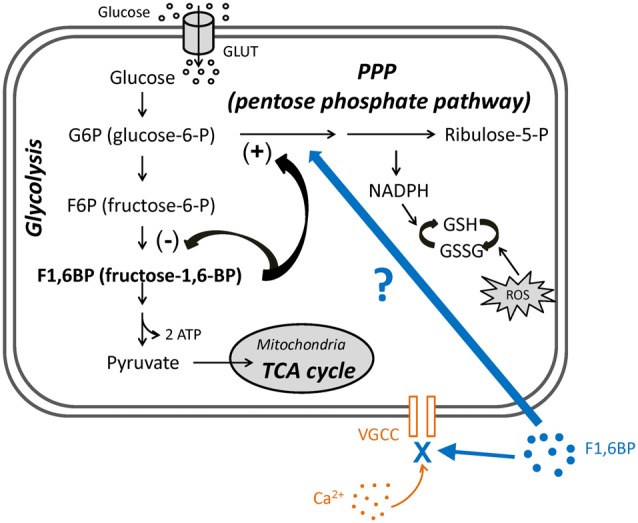
The roles of fructose-1,6-bisphosphate (F1,6BP) in glycolysis and pentose phosphate pathway (PPP) and its proposed extracellular action. Diagram showing the main steps in glycolysis, its relationship to the PPP and the roles of F1,6BP. Glucose enters cytoplasm via glucose transporters (GLUT3 in neurons or GLUT1 in glia), where glycolysis takes place. Glucose is first phosphorylated to glucose-6-P (G6P), is further converted to F1,6BP, and is ultimately catalyzed to pyruvate, which then enters mitochondria to participate in the TCA cycle. G6P also enters the PPP, which generates reduced nicotinamide adenine dinucleotide phosphate (NADPH) and glutathione (GSH) to prevent the cell damage caused by reactive oxygen species (ROS). Accumulation of F1,6BP weakly inhibits F6P conversion to F1,6BP, and potently shunts G6P into PPP, leading to the production of GSH. In addition, exogenous F1,6BP may interfere with voltage-gated calcium channels (VGCC) and reduce Ca^2+^ influx, based on evidence in this study.

The pentose phosphate pathway (PPP) is a secondary metabolic pathway that comprises a “branch” off glycolysis. The PPP is stimulated by the glycolytic intermediate, fructose-1,6-bisphosphate (F1,6BP), diverting glycolysis into the PPP, which produces glutathione (GSH), an antioxidant and endogenous anticonvulsant (Abe et al., 2000; Figure 1). Therefore, stimulation of the PPP has the potential to control excessive neuronal activity and seizures. Interestingly, a previous study reported that administration (i.p.) of the PPP stimulator F1,6BP is associated with anticonvulsant effects in animal models (Lian et al., 2007), supporting the hypothesis that PPP may play an important role in regulating seizures. Yet, how PPP alters neural excitability and seizure activity is largely elusive.

In this study, we further examined the role of the PPP in regulating neuronal excitability and seizure activity, by stimulating the PPP with F1,6BP in hippocampal slices. We found that while F1,6BP consistently blocks epileptiform bursts in hippocampal slices, it does not significantly reduce intrinsic neuronal firing or excitatory synaptic transmission. Unexpectedly, F1,6BP directly blocks voltage-activated calcium current (I_Ca_), an important contributor to epileptiform bursts. Thus, F1,6BP exerts its anti-epileptiform action partly through a previously unrecognized acute extracellular action to attenuate Ca^2+^ influx (Figure 1), at least in this *in vitro* seizure model. This novel mechanism of an intracellular metabolite on an extracellular target provides new insight into metabolic control of neural excitability and seizures.

## Materials and Methods

### Ethics Approval Statement

All procedures used in this study were approved by the Institutional Animal Care and Use Committee of Johns Hopkins University.

### Brain Slice Preparation

Hippocampal slices were prepared from Sprague-Dawley rats (Envigo International, Indianapolis, IN, USA) aged 8–16 days, when they are most susceptible to epileptiform bursting activity in slice electrophysiology experiments (Swann and Brady, 1984). Rat pups were deeply anesthetized by isoflurane inhalation and decapitated. Their brains were quickly removed and placed in pre-chilled and oxygenated low Ca^2+^/high Mg^2+^ cutting solution containing (in mM): 125 NaCl, 3 KCl, 1.25 NaH_2_PO_4_, 25 NaHCO_3_, 0.25 CaCl_2_, 10 MgSO_4_ and 11 glucose, for ~1–2 min. One hemisphere was glued onto the platform of a specimen syringe and embedded with 1.6% low-melting point agarose (type I-B), and placed into a buffer tank filled with cutting solution. Coronal hippocampal slices (350 μm) were cut using a VF-300 compresstome (Precisionary Instruments Inc., Greenville, NC, USA), and transferred to a storage chamber filled with a holding solution (Feldmeyer et al., 2006) containing (in mM): 125 NaCl, 3 KCl, 1.25 NaH_2_PO_4_, 25 NaHCO_3_, 0.5 CaCl_2_, 5 MgSO_4_ and 11 glucose, and continuously bubbled with 95% O_2_ and 5% CO_2_. Slices were allowed to recover for ~2 h, first at 34°C for ~45 min and thereafter at room temperature.

### Electrophysiology

For recording, hippocampal slices were transferred to a submerged chamber and perfused with oxygenated artificial cerebrospinal fluid (aCSF) containing (in mM): 125 NaCl, 3 KCl, 1.25 NaH_2_PO_4_, 25 NaHCO_3_, 1.3 CaCl_2_, 1.3 MgSO_4_ and 11 glucose, at 2 ml/min. For experiments eliciting epileptiform bursts and NMDA currents (see below), Mg^2+^ was omitted from aCSF and Ca^2+^ was elevated to 2 mM. All recordings were conducted at 32–33°C through a digital temperature control system (Scientifica, East Sussex, UK). To visualize and approach individual neurons, a PatchPro 6000 patch-clamp recording system (Scientifica Instruments, East Sussex, UK) was used. This system is comprised of a custom designed motorized microscope with infrared differential interference contrast (IR-DIC) components, light-emitting diode (LED) IR and fluorescence light sources, 40× water immersion objective and CCD camera, and two motorized micromanipulators sitting on top of a motorized movable base plate. The entire visualized patch-clamp procedure was monitored on a computer screen and operated remotely through a control panel and LinLab software (Scientifica Instruments, East Sussex, UK). Electrophysiological signals were acquired by a MultiClamp 700B amplifier, a Digidata-1550 digitizer, and Clampex 10.4 software (Molecular Devices, Sunnyvale, CA, USA). Single or dual whole-cell recordings, extracellular field-potential recording, or simultaneous field potential-whole cell recordings were conducted in hippocampal CA3. In some field-potential experiments, two slices were recorded simultaneously. Synaptic stimulation was delivered by a concentric bipolar electrode (FHC, Bowdoin, ME, USA) placed in the CA3 stratum radiatum 100–200 μm lateral to the recording electrodes, through a digital stimulus generator (STG 4002, Multichannel Systems, Reutlingen, Germany).

Recording pipettes were pulled from filamented thick-walled (outer diameter 1.5 mm, inner diameter 0.86 mm) borosilicate glass through a P-1000 pipette puller (Sutter Instruments, Novato, CA, USA). The electrodes typically have a resistance of 4–9 MΩ. For whole-cell current-clamp experiments, pipettes were filled with K-gluconate-based internal solution containing (in mM): 130 K-gluconate, 10 HEPES, 5.5 EGTA, 0.5 CaCl_2_, 1 NaCl, 2 KCl, 1 MgCl_2_, 10 phosphocreatine-tris, 0.5 Na-GTP, 2 Mg-ATP. The pH was adjusted to 7.25 with 5 M KOH. For whole-cell voltage-clamp experiments, pipettes were filled with Cs-methanesulfonate-based internal solution containing (in mM): 120 Cs-methanesulfonate, 10 HEPES, 5.5 EGTA, 0.5 CaCl_2_, 5 NaCl, 10 phosphocreatine-tris, 0.5 Na-GTP, 4 Mg-ATP supplemented with Na^+^ channel blocker QX 314 (5 mM). The pH was adjusted to 7.25 with CsOH. The estimated liquid junction potentials between the bath medium and the K-gluconate-based or Cs-methanesulfonate-based pipette solution were 7 mV and 10 mV, respectively (calculated using junction potential calculator in PClamp 10, Molecular Devices, Sunnyvale, CA, USA), and were not corrected in the values presented in this article. For field-potential recordings, pipettes were filled with aCSF.

Population epileptiform bursts in CA3 were induced by Mg^2+^-free medium containing 50 μM 4-aminopyridine (4-AP), and recorded extracellularly in non-clamp mode (*I* = 0) with a gain of 100–200. Intrinsic firing of CA3 neurons was recorded in the whole-cell current-clamp mode and in the presence of AMPA-, NMDA- and GABA_A_- receptor antagonists DNQX (10 μM), AP-5 (50 μM) and SR95531 (10 μM), to eliminate synaptic influences. Glutamatergic synaptic currents were evoked by electrical stimulation and recorded in whole-cell voltage-clamp mode holding at −65 mV. Stimuli were delivered at 0.05 Hz at an intensity of 150–300 μA, 100 μs, which was determined as 2–3 times threshold stimulation, defined as the minimal current stimulation (~50–75 μA) to evoke an EPSC. AMPAR-mediated excitatory postsynaptic current (EPSC_AMPA_) was recorded in the presence of SR95531 (10 μM) and AP-5 (50 μM), while NMDAR-mediated excitatory postsynaptic current (EPSC_NMDA_) was evoked in Mg^2+^-free medium and in the presence of SR95531 (10 μM) and DNQX (10 μM). GABA_A_R-mediated current was evoked by local stimulation in the CA3 pyramidal cell layer to directly activated GABAergic neurons, and recorded in the presence of DNQX (10 μM) and AP-5 (50 μM) and held at 0 mV. Voltage-activated calcium current (I_Ca_) was recorded in whole-cell voltage-clamp mode in the presence of SR95531 (10 μM), DNQX (10 μM) and AP-5 (50 μM). A ramp voltage command (−70 to +20 mV, 200–500 ms) and a series of stepwise voltage commands (−70 to +20 mV, 200–500 ms, step: 10-mV) were used to characterize voltage-dependent activation kinetics. For steady-state inactivation kinetics, the membrane potential was first clamped at different levels (−70 to +20 mV) for 1 s (i.e., pre-pulses) and then stepped to −10 mV (200 ms, test pulses). High voltage-activated I_Ca_ was evoked by a single voltage step from −70 mV to −10 mV (200–500 ms), repeated at 0.1 Hz. The series resistance (Rs) was compensated by 55%–75% and I_Ca_ was leak subtracted online using a P4 subtraction paradigm in Clampex 10 (Molecular Devices, Sunnyvale, CA, USA). In a subset of experiments, neurons were loaded with F1,6BP (5 mM) intracellularly via recording pipettes by adding F1,6BP to the internal solution. All signals were acquired at 10 kHz and low-pass filtered at 2 kHz. For analysis, field-potential population bursts were high-pass filtered at 1 Hz offline.

### Data Analysis

Qualitative and quantitative data analyses were conducted using Clampfit 10.4 (Molecular Devices, Sunnyvale, CA, USA), SigmaPlot 11 (SPSS Inc., Chicago, IL, USA), and Excel (Microsoft Corp., WA, USA) programs. The mean amplitude of epileptiform population bursts, EPSC_AMPA_ and EPSC_NMDA_, GABA_A_ current and I_Ca_ and the mean frequency of neuronal firing were calculated from a 5-min recording before and after application of F1,6BP, and averaged across the recorded cells/slices in their respective groups. One-way analyses of variance (ANOVA) was used for statistical analysis across multiple groups, followed by the Holm-Sidak test for comparison between each two groups. Student’s *t*-test was used for comparisons between two groups. Data are presented as means ± SEM with statistical significance set at *p* < 0.05.

### Pharmacological Agents and Chemicals

All chemicals used in this study were purchased from Sigma-Aldrich (St. Louis, MO, USA), except DNQX (di-sodium salt), which was obtained from Abcam (Cambridge, MA, USA).

## Results

### F1,6BP Rapidly Blocks CA3 Epileptiform Population Bursts

**Figure 2:**
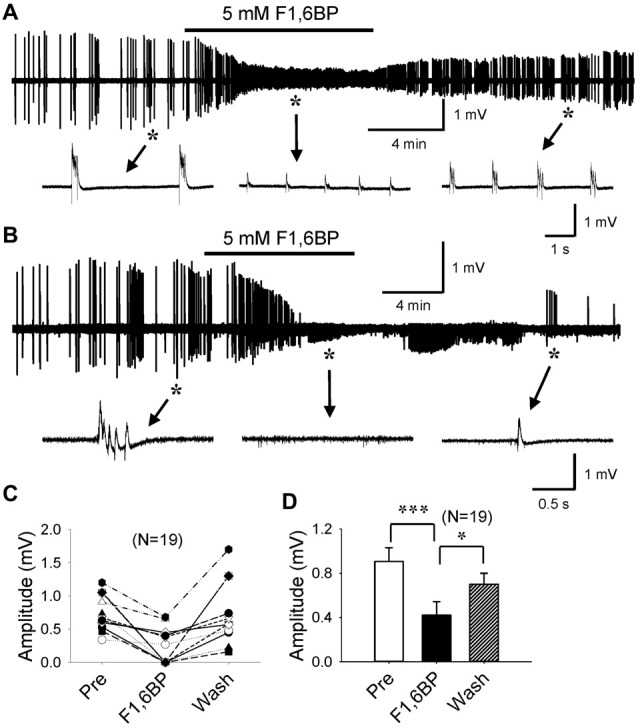
F1,6BP blocks epileptiform population bursts in the CA3 area of hippocampal slices.** (A,B)** Field-potential epileptiform bursts consistently occurred in 0 Mg^2+^ artificial cerebrospinal fluid (aCSF) with 50 μM 4-aminopyridine (4-AP). Bath application of F1,6BP (5 mM) quickly and markedly reduced the amplitude of the bursts **(A)** or completely abolished the bursts **(B)** in ~2/3 of the slices. Bursts recovered when F1,6BP was washed out, which was slow in the case of complete blockade **(B)**. Insets in **(A,B)** indicated by asterisks and arrows showing epileptiform bursts before, during and after F1,6BP application at expanded time scales. Note that single-unit activity was not blocked by F1,6BP (**B**, middle inset). **(C)** Summary data showing the effect of F1,6BP in all tested slices (*n* = 19). **(D)** Averaged burst amplitude before, during and after F1,6BP across slices. *p < 0.05, ***p < 0.001, analyses of variance (ANOVA) followed by the Holm-Sidak test.

F1,6BP has previously been shown to exhibit a prominent anticonvulsant effect in several animal models of acute seizures (Lian et al., 2007). However, no study to date has studied F1,6BP effects on epileptiform activity *in vitro*. We first tested the hypothesis that F1,6BP blocks epileptiform activity in the CA3 area of hippocampal slices. Epileptiform population bursts were consistently induced in 0 Mg^2+^ aCSF with 50 μM 4-AP. Similar to our previous study (Shao and Stafstrom, 2017), these bursts were typically “interictal-like” discharges consisting of 1–4 short bursts, lasting for 100–500 ms and occurring at variable frequencies (Figures 2A,B). Bath application of F1,6BP (2.5–5 mM) markedly suppressed epileptiform bursts in majority (13 of 19, 68%) of the slices (Figures 2A–D). This dosage of F1,6BP is similar to previous *in vitro* studies (3.5 mM, Vexler et al., 2003); 10 mM, (Izumi et al., 2003) and comparable to (but slightly lower than) *in vivo* studies (0.25–1 g/kg, (Lian et al., 2007); 1 g/kg, (Rogido et al., 2003)). More specifically, F1,6BP caused two types suppression: partial and complete. In partial suppression, epileptiform population bursts were minimized in amplitude by F1,6BP but did not disappear, and recovered quickly when F1,6BP was washed out from bathing medium (Figure 2A). In complete suppression, epileptiform bursts were fully abolished by F1,6BP and recovered much more slowly (up to 35 min; Figure 2B). Interestingly, single-unit activity in the slices was not blocked by F1,6BP (Figure 2B, middle inset). Overall, F1,6BP effectively inhibited epileptiform bursts activity in brain slices, corroborating its anticonvulsant effects *in vivo* (Lian et al., 2007). However, the rapid time to blockade with F1,6BP (4.2 ± 0.7 min, Figures 2A,B) was surprising, because it occurred faster than expected for an action mediated via an intracellular metabolic pathway (for comparison, glycolytic inhibition with 2-DG blocks similar epileptiform activity in CA3 in 14.2 ± 1.6 min; Shao and Stafstrom, 2017). Thus, we asked whether the quick F1,6BP effect might be mediated by an acute extracellular mechanism.

### F1,6BP Does Not Decrease Intrinsic Spontaneous Neuronal Firing

**Figure 3:**
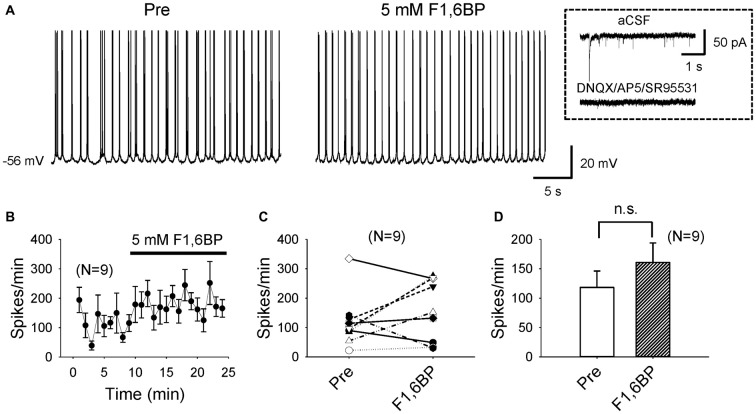
F1,6BP does not stop spontaneous intrinsic firing of CA3 pyramidal neurons.** (A)** Example of intrinsic spontaneous firing in a CA3 pyramidal cell, which was not blocked by F1,6BP (5 mM). These recordings were conducted in the presence of DNQX (10 μM), AP-5 (50 μM) and SR95531 (10 μM), which abolished synaptic events (inset). **(B)** Averaged time course across recorded neurons (*n* = 9) showing the F1,6BP effect on neuronal firing. **(C)** Summary data showing the effect of F1,6BP on individual neurons. **(D)** Averaged spike frequency showing that F1,6BP does not reduce (and tends to increase) intrinsic firing of CA3 pyramidal neurons (*n* = 9). *P* > 0.05, two-tailed paired student’s *t*-test.

To explore cellular mechanisms of F1,6BP on neuronal excitability and seizure susceptibility, we next tested its action on intrinsic firing of CA3 pyramidal neurons, which are known to fire action potentials spontaneously (Wong and Prince, 1978; Hablitz and Johnston, 1981; Traub and Wong, 1983). To minimize synaptic influence on intrinsic neuronal firing due to the dense interconnections between CA3 neurons, particularly at young ages (Miles and Wong, 1986; Shao and Dudek, 2009), we blocked AMPA-, NMDA- and GABA_A_ receptors with DNQX (10 μM), AP-5 (50 μM) and SR95531 (10 μM), respectively. The average resting membrane potential (RMP) of the recorded CA3 neurons was −66 ± 1.3 mV (*n* = 9). Most of these neurons (6/9) fired action potentials spontaneously at their RMP; the other neurons (3/9) were manually depolarized to ~60 mV via current injection to fire action potentials. Under these experimental conditions, the neurons fired action potentials continuously at an average frequency of ~2–3 Hz, mostly in bursts (Figures 3A–D), persisting for prolonged periods (up to >60 min). Bath application of F1,6BP did not stop neuronal firing (Figures 3A–C); in fact, it tended to increase the firing frequency in some neurons (Figures 3B,C). Overall, F1,6BP did not significantly change the frequency of intrinsic firing of CA3 neurons (Figures 3B,D; 118 ± 28 spikes/min vs. 161 ± 33 spikes/min, *p* > 0.05, *n* = 9), suggesting that F1,6BP does not block epileptiform population bursts through inhibition of intrinsic firing mechanisms.

### F1,6BP Does Not Significantly Reduce Glutamatergic Synaptic Transmission

**Figure 4:**
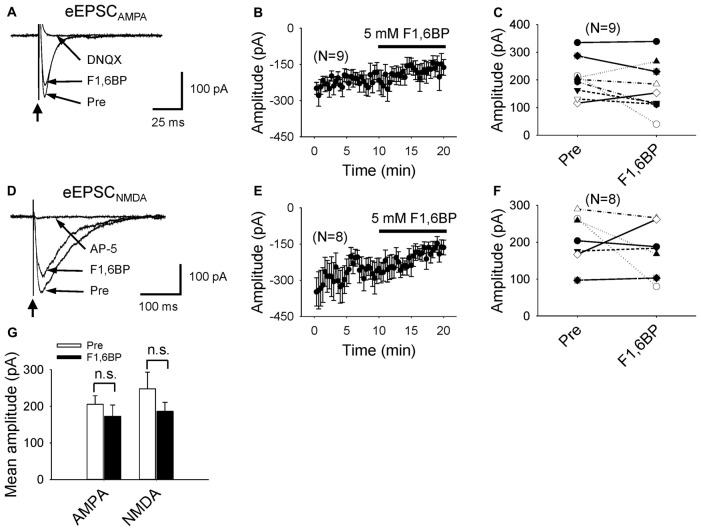
F1,6BP does not block AMPA or NMDA receptor-mediated glutamatergic transmission.** (A)** Superimposed traces showing AMPAR-mediated excitatory postsynaptic current (EPSC_AMPA_) before and during F1,6BP and in DNQX (long arrows). EPSC_AMPA_ was not blocked by F1,6BP (5 mM) but was completely blocked by the AMPAR antagonist, DNQX (10 μM). These currents were evoked by synaptic stimulation (thick vertical arrowhead) in the presence of NMDAR and GABA_A_R antagonists AP-5 (50 μM) and SR95531 (10 μM). Note that each trace is an average of 10 raw current traces. The stimulation artifact was partially removed from the trace for clarity. **(B)** Averaged time course showing the effect of F1,6BP on EPSC_AMPA_ (*n* = 9). **(C)** Summary data showing the effect of F1,6BP on EPSC_AMPA_ on individual neurons. **(D)** Superimposed traces showing NMDAR-mediated excitatory postsynaptic current (EPSC_NMDA_) before, during F1,6BP and in AP-5 (horizontal arrows). EPSC_NMDA_ was slightly reduced in F1,6BP and completely abolished in AP-5 (50 μM). The currents were evoked by synaptic stimulation (vertical arrow) in 0 Mg^2+^ solution and in the presence of AMPAR and GABA_A_R antagonists DNQX (10 μM) and SR95531 (10 μM). Each shown trace is an average of 10 raw current traces. Note that the slow decay of EPSC_NMDA_ compared to that of EPSC_AMPA_. **(E)** Averaged time course of F1,6BP on EPSC_NMDA_ (*n* = 8). **(F)** Effect of F1,6BP on EPSC_NMDA_ on individual neurons. **(G)** Mean amplitudes of EPSC_AMPA_ and EPSC_NMDA_ were not significantly changed by F1,6BP (*p* > 0.05, two tailed paired student’s *t*-test).

Next, we asked whether F1,6BP blocked glutamatergic transmission, and tested its effect on EPSC_AMPA_ and EPSC_NMDA_ separately. EPSC_AMPA_ was evoked in the presence of AP-5 (50 μM) and SR95531 (10 μM) to block NMDA and GABA_A_ receptors. The EPSC_AMPA_ was typically fast (~20 ms) with an amplitude of ~200 pA (Figure 4A). Addition of F1,6BP (5 mM) seemed to slightly reduce the amplitude of the EPSC_AMPA_ but did not cause overall significant reduction (Figures 4A–C,G; 206 ± 23 pA vs. 173 ± 31 pA, *p* > 0.05, *n* = 9). EPSC_NMDA_ was evoked in Mg^2+^-free solution in the presence of DNQX (10 μM) and SR95531 (10 μM) to block AMPA and GABA_A_ receptors, and held at −65 mV. The extracellular Ca^2+^ concentration was modestly elevated to 2 mM (from 1.3 mM) to partially compensate for membrane surface charge of Mg^2+^ (Hille et al., 1975) and reduce multi-synaptic responses. Compared with EPSC_AMPA_, the EPSC_NMDA_ was usually larger and much slower (>100 ms; Figure 4D). Application of F1,6BP tended to decrease the amplitude of EPSC_NMDA_ but the reduction was not significant overall (Figures 4E–G, 248 ± 45 pA vs. 186 ± 24 pA, *p* > 0.05, *n* = 8). These data suggest that it is unlikely that F1,6BP blocks epileptiform bursts through reduction of glutamatergic transmission.

### F1,6BP Reduces GABAergic Synaptic Transmission

**Figure 5:**
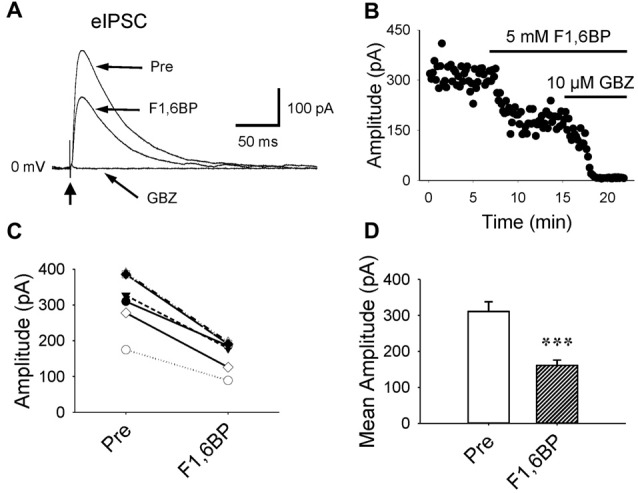
F1,6BP reduces GABA_A_R-mediated synaptic currents.** (A)** Superimposed traces showing GABA_A_R-mediated currents before and during F1,6BP and in SR95531 (GABAzine, GBZ, 10 μM; long arrows). GABA currents were reduced by F1,6BP (5 mM) and completely blocked by the GABA_A_R antagonist, SR95531 (GBZ, 10 μM). These currents were evoked by synaptic stimulation (thick vertical arrowhead) in the presence of AMPAR and NMDAR antagonists DNQX (10 μM) and AP-5 (50 μM) and held at 0 mV (outward currents). Note that each trace is an average of 10 raw current traces and the stimulation artifact was partially removed from the trace for clarity. **(B)** Time course showing the action of F1,6BP and SR95531 (GBZ) on evoked GABA_A_ currents. **(C)** Summary data showing the effect of F1,6BP on individual neurons. **(D)** Mean amplitude of evoked GABA_A_ currents before and after F1,6BP. ***p < 0.001, two-tailed paired *t*-test (*n* = 6).

Further, we examined whether F1,6BP affects GABAergic transmission. GABA_A_R-mediated currents were consistently evoked in the presence of DNQX (10 μM) and AP-5 (50 μM) by holding membrane voltage at 0 mV. Unlike its effect on glutamatergic synapses, bath application of F1,6BP reduced GABA current by ~50% (Figure 5A–D, 311 ± 27 pA vs. 161 ± 15 pA, *p* < 0.001, *n* = 6), suggesting that F1,6BP causes synapse-specific effects.

### F1,6BP Consistently Inhibits Voltage-Activated Calcium Currents (I_Ca_)

**Figure 6:**
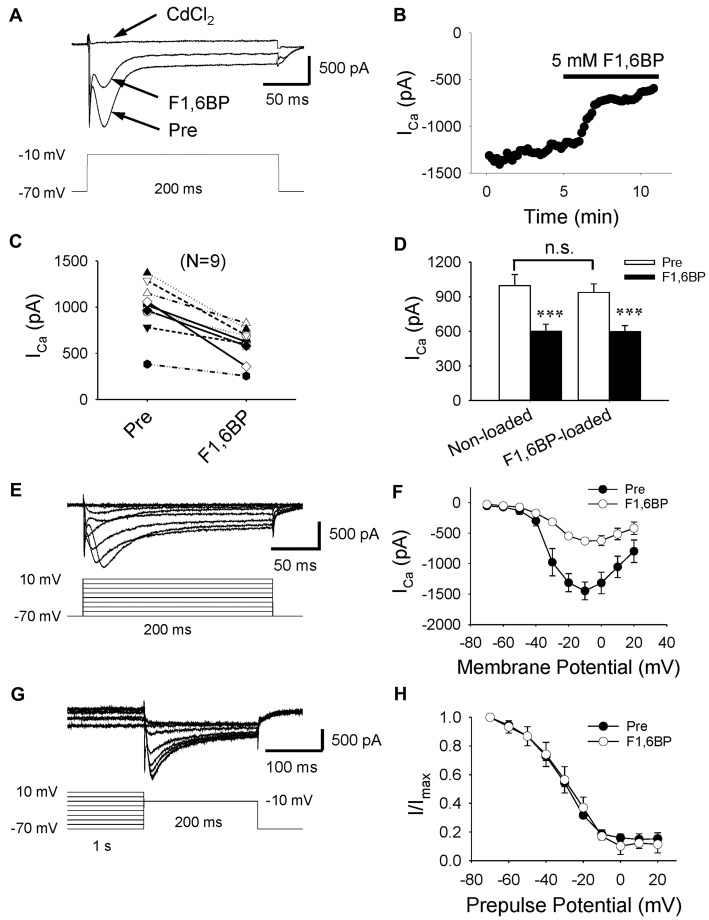
F1,6BP inhibits high voltage-activated calcium currents (I_Ca_).** (A)** Superimposed traces showing I_Ca_ prior to and during F1,6BP application and in the presence of the Ca^2+^ channel blocker CdCl_2_ (horizontal arrows). I_Ca_ was activated by a depolarizing voltage step showing in the lower panel and leak-subtracted online. All recordings were made in the presence of extracellular DNQX (10 μM), AP-5 (50 μM) and SR95531 (10 μM) which block glutamatergic and GABAergic synaptic transmission, and intracellular Cs^+^ (120 mM) and QX314 (5 mM) which blocks K^+^ channels (including GABA_B_ receptors) and Na^+^ channels, respectively. Application of F1,6BP (5 mM) consistently attenuated I_Ca_ by ~40%, and CdCl_2_ (100 μM) completely abolished I_Ca_. **(B)** The time course showing the reduction of I_Ca_ amplitude in the recorded neuron during application of F1,6BP. **(C)** Summary data showing the effect of F1,6BP on I_Ca_ on individual neurons. **(D)** Bar graph showing the overall reduction of I_Ca_ amplitude caused by F1,6BP averaged across non-loaded neurons (*n* = 9) and F1, 6BP-loaded neurons (*n* = 6). ***p < 0.001, paired student’s *t*-test. **(E)** Example of a family of I_Ca_ (upper panel) elicited by a series of depolarizing voltage pulses (lower panel) showing I_Ca_ activation kinetics. **(F)** The current-voltage relationship averaged across all neurons (*n* = 9) showing the voltage-dependence of I_Ca_ before (black circles) and during F1,6BP (open circles). **(G)** Current traces showing the steady-state inactivation of I_Ca_ (upper panel) induced by a series of 1-s depolarizing pre-pulses holding at different voltage levels (lower panel). **(H)** Plots of normalized current amplitudes against pre-pulse holding voltage showing the inactivation kinetics of I_Ca_, which were not changed by F1,6BP.

Given that two of the main contributors to epileptiform bursts, intrinsic neuronal firing and synaptic transmission, were not significantly altered by F1,6BP, it was reasonable to speculate that F1,6BP somehow acts through I_Ca_, which is known to be essential for the generation of epileptiform bursts (Schwartzkroin and Wyler, 1980; Kohling et al., 2000; Siwek et al., 2012). To test this hypothesis, we evoked I_Ca_ in CA3 neurons using depolarizing voltage protocols (Figures 6A,E). I_Ca_ began to be activated at ~−40 mV and peaked at ~−10 mV (Figures 6E,F). I_Ca_ appeared to comprise a prominent inactivating component followed by a smaller steady-state component, and was completely abolished by the non-specific Ca^2+^ channel blocker, CdCl_2_ (100 μM; Figures 6A,E). These data are consistent with a previous study that showed that immature CA3 neurons have significantly more prominent inactivating I_Ca_ than adult neurons (Thompson and Wong, 1991). The peak amplitude of I_Ca_ ranged from 400 pA to 1400 pA (on average: 996 pA, *n* = 9). Application of F1,6BP (5 mM) quickly (2–3 min) and consistently attenuated I_Ca_ by ~40% (Figures 6A–D, 996 ± 97 pA vs. 600 ± 62 pA, *p* < 0.001, *n* = 9). To test the whether F1,6BP can block I_Ca_ intracellularly, a subset of CA3 neurons were loaded with F1,6BP (5 mM) intracellularly through recording pipettes. In these neurons, the amplitude of I_Ca_ activated by the same protocol was similar to that in non-loaded neurons (Figure 6D, 938 ± 91 pA, *n* = 6; vs. 996 ± 97 pA, *n* = 9; *p* > 0.05). Moreover, further extracellular application of F1,6BP caused similar reduction of I_Ca_ in the F1, 6BP-loaded neuron (~40%, Figure 6D, 938 ± 91 vs. 598 ± 64, *p* < 0.001, *n* = 6). These data suggest that F1,6BP most likely blocks I_Ca_ extracellularly rather than intracellularly. F1,6BP did not seem to alter the voltage dependence of I_Ca_ as it was activated at the same voltage range before and during F1,6BP application (Figure 6F). Steady-state inactivation, assessed by the amplitude of I_Ca_ after 1 s-long pre-pulses holding at different voltage levels (Figure 6G), was also not altered by F1,6BP. In fact, the inactivation kinetics of I_Ca_ before and during F1,6BP were almost identical (Figure 6H). Altogether, these data suggest that F1,6BP blocks a significant portion of I_Ca_, which may account for its anticonvulsant action on epileptiform bursts.

### Epileptiform Bursts in CA3 Are Ca^2+^ Dependent

**Figure 7:**
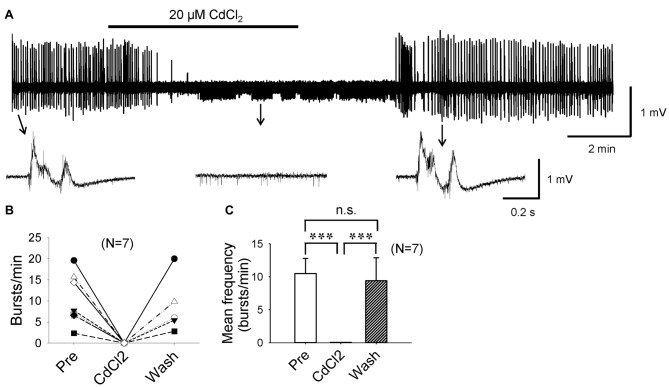
Epileptiform population bursts in CA3 are Ca^2+^-dependent. **(A)** Example showing that field-potential epileptiform bursts in CA3 occurred in 0 Mg^2+^ and 50 μM 4-AP solution were completely abolished by low concentration (20 μM) of CdCl_2_, a non-selective Ca^2+^ channel blocker. When CdCl_2_ was washed out from the bath, epileptiform bursts recovered fully. The epileptiform bursts before, during, and after CdCl_2_ application indicated by arrows are shown in expanded time scale in insets below. Note that similar to F1,6BP, CdCl_2_ did not block single-unit activity in the slice (middle inset). **(B)** Summary data showing the effect of CdCl_2_ on burst frequency in individual slices. **(C)** Bar graph showing the overall effect of CdCl_2_ across all slices. ***p < 0.001, ANOVA (*n* = 7).

If it were true that F1,6BP blocks epileptiform bursts by reducing I_Ca_, it would be expected that Ca^2+^ channel antagonists would also block epileptiform bursts as F1,6BP did. To confirm this, we applied CdCl_2_, a non-selective Ca^2+^ channel antagonist. As expected, a low concentration of CdCl_2_ (20 μM) completely and reversibly blocked epileptiform bursts (Figures 7A–C). Similar to F1,6BP, CdCl_2_ did not block the single-unit activities in the slices (Figures 2B, 7A, middle insets). Taken together, these data confirm that the epileptiform bursts in CA3 are Ca^2+^-dependent and can be blocked by Ca^2+^ channel blockers or modulators (such as F1,6BP).

## Discussion

The main findings of the present study are: (1) the glucose metabolite and PPP stimulator, F1,6BP, effectively blocks epileptiform activity in hippocampal slices; (2) F1,6BP does not stop intrinsic neuronal firing or significantly reduce AMPAR or NMDAR-mediated synaptic currents; (3) F1,6BP causes a reduction of GABA_A_R-mediated synaptic currents; (4) F1,6BP consistently attenuates I_Ca_; and (5) epileptiform bursts in this model are Ca^2+^ dependent. To our knowledge, this is the first examination of the role of PPP in seizure reduction and the anticonvulsant action of F1,6BP in brain slices, focusing on underlying cellular mechanisms.

### Novel Mechanism of F1,6BP’s Anti-Seizure Activity

Our data in hippocampal slices corroborates the pioneering work showing that F1,6BP inhibits seizures in animal models (Lian et al., 2007). As in that study (Lian et al., 2007), we initially hypothesized that the anti-seizure activity of F1,6BP was mediated by an intracellular metabolic effect of shifting glycolysis to PPP (Figure 1), thus promoting the production of the endogenous antioxidant and anticonvulsant, GSH (Lian et al., 2007). Yet, we noticed that the time to block epileptiform bursts by F1,6BP (4.2 ± 0.7 min) was much quicker than epileptiform bursts blocked by the glycolytic inhibitor 2-DG in our previous study (14.2 ± 1.6 min; Shao and Stafstrom, 2017). The short time to blockade suggested that F1,6BP might have a direct extracellular effect independent of its assumed intracellular effect and prompted us to examine its effect on extracellular mechanisms that contribute to neural excitability. However, F1,6BP did not stop neuronal firing nor did it significantly reduce excitatory synaptic transmission, two of the major contributors to epileptiform bursting (Traub and Wong, 1983). Finally and unexpectedly, we discovered that F1,6BP consistently blocks a significant portion of voltage-activated I_Ca_ extracellularly. Since epileptiform bursts in CA3 are Ca^2+^-dependent and readily blocked by Ca^2+^ channel blockade (CdCl_2_), these data strongly suggest that F1,6BP achieves its anticonvulsant activity in part by attenuating Ca^2+^ influx, at least in our *in vitro* seizure model.

Epileptiform bursts may be mediated by both presynaptic and postsynaptic Ca^2+^-dependent mechanisms. Presynaptic Ca^2+^ influx is important for transmitter release. Postsynaptic (somatic) I_Ca_ is known to be essential for the genesis and maintenance the plateau potentials of epileptiform bursts (Schwartzkroin and Wyler, 1980; Kohling et al., 2000; Siwek et al., 2012). Our data that F1,6BP did not cause significant reduction in EPSCs suggest that F1,6BP blocks epileptiform bursts mainly by reducing postsynaptic (somatic) rather than presynaptic Ca^2+^ influx; and that a moderate reduction (40%) of postsynaptic Ca^2+^ influx is sufficient to block seizures_._ The 40% reduction of Ca^2+^ influx is derived from somatic high-voltage activated (HVA) Ca^2+^ currents. Whether presynaptic Ca^2+^ entry is similarly reduced by F1,6BP is unclear. Possibly, Ca^2+^ channels at presynaptic terminals of glutamatergic synapses may be less sensitive to F1,6BP or F1,6BP might block certain types Ca^2+^ channels that are not coupled to glutamate release (Turner et al., 1992; Dunlap et al., 1995). In contrast, Ca^2+^ influx at the GABAergic presynaptic terminals is likely affected by F1,6BP as indicated by the reduction of GABA_A_ current. These data suggest that F1,6BP differentially affects glutamatergic and GABAergic transmission. At the developmental period in this study (P8–16), the action of GABA on CA3 neurons is believed to have switched from excitatory (P0–P6) to inhibitory (Ben-Ari et al., 1990). However, the roles of GABAergic inhibition in seizures are complex. Recent studies have demonstrated that selective activation of perisomatic targeting interneurons (such as parvalbumin-expressing neurons) leads to initiation of seizures (Toyoda et al., 2015; Yekhlef et al., 2015; Khoshkhoo et al., 2017), while activation of axonic targeting interneurons (or axon-axonic synapses) inhibits seizures (Wang et al., 2014). In this study, we stimulated the CA3 pyramidal layer, where the axons of perisomatic interneurons are located, thus the resulting IPSCs are mostly derived from perisomatic synapses. A reduction in the strength of this type of inhibition by F1,6BP might therefore contribute to the reduction of epileptiform activity. Whether F1,6BP exerts additional effects on intracellular metabolic pathways in our model remains to be determined. Apparently, the mechanisms underlying the anticonvulsant action of F1,6BP in animal models (Lian et al., 2007) are likely to be more complex than in brain slices. The extent to which this extracellular mechanism contributes to F1,6BP’s *in vivo* anticonvulsant action is uncertain. Arguably, administration of antioxidants does not consistently block seizures in the same animal models (Xu and Stringer, 2008), which suggests that F1,6BP may act on other targets in addition to PPP activation and GSH production. Nonetheless, the present study revealed a novel extracellular mechanism of F1,6BP that may explain its anticonvulsant activity, at least in this *in vitro* model of seizures.

CA3 neurons in the developing hippocampus possess both HVA and low-voltage activated (LVA) Ca^2+^ channels (Thompson and Wong, 1991). It is generally recognized that Ca^2+^ influx through HVA channels (particularly L- and R-types) are critically involved in the genesis of the plateau potential of epileptiform bursts (Straub et al., 2000; Siwek et al., 2012), while Ca^2+^ influx through LVA channels (i.e., T-type) contributes to rebound bursting and pacemaking activity (Siwek et al., 2012). The major component of I_Ca_ in the present study resembles the HVA inactivating currents (R, N-, P/Q) with an activation threshold of −40 mV and fast decay kinetics (Yamakage and Namiki, 2002; Lacinová, 2005; Catterall, 2011), mixed with a smaller component of non-inactivating current (L-type). This is consistent with a previous study showing that inactivating I_Ca_ contributed to a significantly larger percentage of the total I_Ca_ in immature than adult CA3 neurons (Thompson and Wong, 1991). F1,6BP reduces the amplitude of I_Ca_ without altering channel activation and inactivation kinetics, suggesting that F1,6BP does not bind to HVA Ca^2+^ channels and works in a way that is different from conventional Ca^2+^ channel blockers. We did not further differentiate which specific subtypes of HVA Ca^2+^ channel are blocked by F1,6BP nor did we evaluate the effect of F1,6BP on LVA (T-type) I_Ca_, which is beyond the main focus of the present study and may be addressed in future studies. Alternatively, F1,6BP may reduce Ca^2+^ influx by chelating Ca^2+^ ions because of its negative charges (Hassinen et al., 1991). A reduction of Ca^2+^ influx may also affect neuronal firing (by reducing Ca^2+^-activated K^+^ currents). Our data show that F1,6BP tends to increase neuronal firing but this effect did not reach statistical significance.

F1,6BP has previously been shown to be neuroprotective against hypoxic-ischemic injury (Farias et al., 1990; Kelleher et al., 1995; Sola et al., 1996) and excitotoxic injury (Rogido et al., 2003) by stabilizing intracellular Ca^2+^ mediated by activation of phospholipase C (PLC; Donohoe et al., 2001; Fahlman et al., 2002; Rogido et al., 2003). Whether this mechanism occurs in seizures is not known. Also, we do not completely rule out the possibility that F1,6BP acts through an intracellular metabolic pathway (i.e., PPP), in addition to its action on Ca^2+^ channels. Theoretically, with its negative charge and lack of known transporter, it may be difficult for exogenous F1,6BP to enter cells, but several studies have shown a specific membrane permeability for F1,6BP (Hardin and Roberts, 1994; Wheeler et al., 2004; Wheeler and Chien, 2012).

### Metabolic Control of Neural Excitability and Metabolic Substrates as Novel Antiepileptic Drugs

Manipulation of neurometabolism exerts broad action and mechanisms influencing neural excitability and seizures. For instance, the anti-seizure effects of the KD may involve activation of adenosine receptors (Kawamura et al., 2010, 2016) and K_ATP_ (Ma et al., 2007; Lutas and Yellen, 2013), production of ketone bodies that inhibit transmitter release (Juge et al., 2010) or alteration of mitochondrial activity (Kim et al., 2015). Glycolytic inhibition with 2-DG not only blocks acute epileptiform activity in brain slices and animal models (Stafstrom et al., 2009; Forte et al., 2016; Shao and Stafstrom, 2017), but also interrupts the chronic process of epileptogenesis (Garriga-Canut et al., 2006; Stafstrom et al., 2009). Other metabolic interventions include disruption of the glial-neuronal energy coupling by targeting the so-called “astrocyte-neuron lactate shuttle” (Belanger et al., 2011) which also effectively blocks seizure activity (Sada et al., 2015). The efficacy of anticonvulsant activity of F1,6BP first reported by Stringer and colleagues and Lian et al. (2007) was quite remarkable in that it not only blocked seizures in all three animal models tested (KA, pilocarpine, PTZ), but also exceeded the treatment efficacy of 2-DG, valproic acid and KD. Therefore, F1,6BP appears to be a promising antiepileptic agent. The present study reveals an unexpected extracellular effect of F1,6BP, effectively blocking seizures by attenuating I_Ca_ without disrupting normal physiological function (neuronal firing and excitatory synaptic transmission), which is desirable for clinical anti-seizure treatment. Moreover, its neuroprotective effect against hypoxic-ischemic injury would be additionally beneficial to patients, as seizures are often accompanied by or caused by hypoxic-ischemic injury, particularly in neonates (Kang and Kadam, 2015). Also, it is important to note that as a natural intracellular glucose metabolite, F1,6BP exerts very little toxicity even at a dose eight times larger than its effective dose (Vexler et al., 1999). All these features of F1,6BP makes it an attractive candidate for an effective and safe antiepileptic agent.

In summary, metabolic interventions represent a novel strategy for treatment of drug-refractory seizures. We have identified a novel mechanism of the promising metabolic anticonvulsant, F1,6BP, which may provide new insight into its anti-seizure and/or neuroprotective activity.

## Author Contributions

L-RS and CS conceptualized the study and wrote the manuscript; L-RS designed the research and conducted the experiments, and analyzed data; and GW contributed to experiments.

## Conflict of Interest Statement

The authors declare that the research was conducted in the absence of any commercial or financial relationships that could be construed as a potential conflict of interest.

## References

[B1] Abe K. Nakanishi K. Saito H. (2000). The possible role of endogenous glutathione as an anticonvulsant in mice. Brain Res. 854, 235–238. 10.1016/s0006-8993(99)02269-6 10784128

[B2] Ames A. III. (2000). CNS energy metabolism as related to function. Brain Res. Brain Res. Rev. 34, 42–68. 10.1016/s0165-0173(00)00038-2 11086186

[B3] Belanger M. Allaman I. Magistretti P. J. (2011). Brain energy metabolism: focus on astrocyte-neuron metabolic cooperation. Cell Metab. 14, 724–738. 10.1016/j.cmet.2011.08.016 22152301

[B4] Ben-Ari Y. Rovira C. Gaiarsa J. L. Corradetti R. Robain O. Cherubini E. (1990). Gabaergic mechanisms in the CA3 hippocampal region during early postnatal life. Prog. Brain Res. 83, 313–321. 10.1016/s0079-6123(08)61259-5 2168059

[B5] Catterall W. A. (2011). Voltage-gated calcium channels. Cold Spring Harb. Perspect. Biol. 3:a003947. 10.1101/cshperspect.a003947 21746798 PMC3140680

[B6] Donohoe P. H. Fahlman C. S. Bickler P. E. Vexler Z. S. Gregory G. A. (2001). Neuroprotection and intracellular Ca^2+^ modulation with fructose-1,6-bisphosphate during *in vitro* hypoxia-ischemia involves phospholipase C-dependent signaling. Brain Res. 917, 158–166. 10.1016/s0006-8993(01)02849-9 11640901

[B7] Dunlap K. Luebke J. I. Turner T. J. (1995). Exocytotic Ca^2+^ channels in mammalian central neurons. Trends Neurosci. 18, 89–98. 10.1016/0166-2236(95)93882-x 7537420

[B8] Fahlman C. S. Bickler P. E. Sullivan B. Gregory G. A. (2002). Activation of the neuroprotective ERK signaling pathway by fructose-1,6-bisphosphate during hypoxia involves intracellular Ca^2+^ and phospholipase C. Brain Res. 958, 43–51. 10.1016/s0006-8993(02)03433-9 12468029

[B9] Farias L. A. Smith E. E. Markov A. K. (1990). Prevention of ischemic-hypoxic brain injury and death in rabbits with fructose-1,6-diphosphate. Stroke 21, 606–613. 10.1161/01.str.21.4.606 2326842

[B10] Feldmeyer D. Lübke J. Sakmann B. (2006). Efficacy and connectivity of intracolumnar pairs of layer 2/3 pyramidal cells in the barrel cortex of juvenile rats. J. Physiol. 575, 583–602. 10.1113/jphysiol.2006.105106 16793907 PMC1819447

[B11] Forte N. Medrihan L. Cappetti B. Baldelli P. Benfenati F. (2016). 2-Deoxy-D-glucose enhances tonic inhibition through the neurosteroid-mediated activation of extrasynaptic gabaa receptors. Epilepsia 57, 1987–2000. 10.1111/epi.13578 27735054

[B12] Freeman J. M. Vining E. P. (1998). Ketogenic diet: a time-tested, effective, and safe method for treatment of intractable childhood epilepsy. Epilepsia 39, 450–451. 10.1111/j.1528-1157.1998.tb01400.x 9578037

[B13] Garriga-Canut M. Schoenike B. Qazi R. Bergendahl K. Daley T. J. Pfender R. M.. (2006). 2-Deoxy-D-glucose reduces epilepsy progression by nrsf-ctbp-dependent metabolic regulation of chromatin structure. Nat. Neurosci. 9, 1382–1387. 10.1038/nn1791 17041593

[B14] Hablitz J. J. Johnston D. (1981). Endogenous nature of spontaneous bursting in hippocampal pyramidal neurons. Cell. Mol. Neurobiol. 1, 325–334. 10.1007/bf00716267 6765736 PMC11572844

[B15] Hardin C. D. Roberts T. M. (1994). Metabolism of exogenously applied fructose 1,6-bisphosphate in hypoxic vascular smooth muscle. Am. J. Physiol. 267, H2325–H2332. 10.1152/ajpheart.1994.267.6.h2325 7810732

[B16] Hassinen I. E. Nuutinen E. M. Ito K. Nioka S. Lazzarino G. Giardina B.. (1991). Mechanism of the effect of exogenous fructose 1,6-bisphosphate on myocardial energy metabolism. Circulation 83, 584–593. 10.1161/01.cir.83.2.584 1991376

[B17] Hille B. Ritchie J. M. Strichartz G. R. (1975). The effect of surface charge on the nerve membrane on the action of tetrodotoxin and saxitoxin in frog myelinated nerve. J. Physiol. 250, 34P–35P. 1177130

[B18] Izumi Y. Benz A. M. Katsuki H. Matsukawa M. Clifford D. B. Zorumski C. F. (2003). Effects of fructose-1,6-bisphosphate on morphological and functional neuronal integrity in rat hippocampal slices during energy deprivation. Neuroscience 116, 465–475. 10.1016/s0306-4522(02)00661-9 12559101

[B19] Juge N. Gray J. A. Omote H. Miyaji T. Inoue T. Hara C.. (2010). Metabolic control of vesicular glutamate transport and release. Neuron 68, 99–112. 10.1016/j.neuron.2010.09.002 20920794 PMC2978156

[B20] Kang S. K. Kadam S. D. (2015). Neonatal seizures: impact on neurodevelopmental outcomes. Front. Pediatr. 3:101. 10.3389/fped.2015.00101 26636052 PMC4655485

[B22] Kawamura M. Jr. Ruskin D. N. Masino S. A. (2010). Metabolic autocrine regulation of neurons involves cooperation among pannexin hemichannels, adenosine receptors and katp channels. J. Neurosci. 30, 3886–3895. 10.1523/JNEUROSCI.0055-10.2010 20237259 PMC2872120

[B21] Kawamura M. Jr. Ruskin D. N. Masino S. A. (2016). Metabolic therapy for temporal lobe epilepsy in a dish: investigating mechanisms of ketogenic diet using electrophysiological recordings in hippocampal slices. Front. Mol. Neurosci. 9:112. 10.3389/fnmol.2016.00112 27847463 PMC5088211

[B23] Kelleher J. A. Chan P. H. Chan T. Y. Gregory G. A. (1995). Energy metabolism in hypoxic astrocytes: protective mechanism of fructose-1,6-bisphosphate. Neurochem. Res. 20, 785–792. 10.1007/bf00969690 7477671

[B24] Khoshkhoo S. Vogt D. Sohal V. S. (2017). Dynamic, cell-type-specific roles for GABAergic interneurons in a mouse model of optogenetically inducible seizures. Neuron 93, 291–298. 10.1016/j.neuron.2016.11.043 28041880 PMC5268075

[B25] Kim D. Y. Simeone K. A. Simeone T. A. Pandya J. D. Wilke J. C. Ahn Y.. (2015). Ketone bodies mediate antiseizure effects through mitochondrial permeability transition. Ann. Neurol. 78, 77–87. 10.1002/ana.24424 25899847 PMC4480159

[B26] Kohling R. Straub H. Speckmann E. J. (2000). Differential involvement of l-type calcium channels in epileptogenesis of rat hippocampal slices during ontogenesis. Neurobiol. Dis. 7, 471–482. 10.1006/nbdi.2000.0300 10964616

[B27] Lacinová L. (2005). Voltage-dependent calcium channels. Gen. Physiol. Biophys. 24, 1–78. 16096350

[B28] Lian X. Y. Khan F. A. Stringer J. L. (2007). Fructose-1,6-bisphosphate has anticonvulsant activity in models of acute seizures in adult rats. J. Neurosci. 27, 12007–12011. 10.1523/JNEUROSCI.3163-07.2007 17978042 PMC6673383

[B29] Lutas A. Yellen G. (2013). The ketogenic diet: metabolic influences on brain excitability and epilepsy. Trends Neurosci. 36, 32–40. 10.1016/j.tins.2012.11.005 23228828 PMC3534786

[B30] Ma W. Berg J. Yellen G. (2007). Ketogenic diet metabolites reduce firing in central neurons by opening K_ATP_ channels. J. Neurosci. 27, 3618–3625. 10.1523/JNEUROSCI.0132-07.2007 17409226 PMC6672398

[B31] Magistretti P. J. Pellerin L. (1996). Cellular bases of brain energy metabolism and their relevance to functional brain imaging: evidence for a prominent role of astrocytes. Cereb. Cortex 6, 50–61. 10.1093/cercor/6.1.50 8670638

[B32] Miles R. Wong R. K. (1986). Excitatory synaptic interactions between ca3 neurones in the guinea-pig hippocampus. J. Physiol. 373, 397–418. 10.1113/jphysiol.1986.sp016055 3018233 PMC1182545

[B33] Rogido M. Husson I. Bonnier C. Lallemand M. C. Merienne C. Gregory G. A.. (2003). Fructose-1,6-biphosphate prevents excitotoxic neuronal cell death in the neonatal mouse brain. Dev. Brain Res. 140, 287–297. 10.1016/s0165-3806(02)00615-6 12586434

[B34] Sada N. Lee S. Katsu T. Otsuki T. Inoue T. (2015). Epilepsy treatment. Targeting LDH enzymes with a stiripentol analog to treat epilepsy. Science 347, 1362–1367. 10.1126/science.aaa1299 25792327

[B35] Schwartzkroin P. A. Wyler A. R. (1980). Mechanisms underlying epileptiform burst discharge. Ann. Neurol. 7, 95–107. 10.1002/ana.410070202 6245619

[B36] Shao L. R. Dudek F. E. (2009). Both synaptic and intrinsic mechanisms underlie the different properties of population bursts in the hippocampal ca3 area of immature versus adult rats. J. Physiol. 587, 5907–5923. 10.1113/jphysiol.2009.179887 19884320 PMC2808548

[B37] Shao L. R. Stafstrom C. E. (2017). Glycolytic inhibition by 2-deoxy-d-glucose abolishes both neuronal and network bursts in an *in vitro* seizure model. J. Neurophysiol. 118, 103–113. 10.1152/jn.00100.2017 28404824 PMC5494374

[B38] Siwek M. Henseler C. Broich K. Papazoglou A. Weiergräber M. (2012). Voltage-gated Ca^2+^ channel mediated Ca^2+^ influx in epileptogenesis. Adv. Exp. Med. Biol. 740, 1219–1247. 10.1007/978-94-007-2888-2_55 22453990

[B39] Sola A. Berrios M. Sheldon R. A. Ferriero D. M. Gregory G. A. (1996). Fructose-1,6-bisphosphate after hypoxic ischemic injury is protective to the neonatal rat brain. Brain Res. 741, 294–299. 10.1016/s0006-8993(96)00984-5 9001735

[B41] Stafstrom C. E. Ockuly J. C. Murphree L. Valley M. T. Roopra A. Sutula T. P. (2009). Anticonvulsant and antiepileptic actions of 2-deoxy-d-glucose in epilepsy models. Ann. Neurol. 65, 435–447. 10.1002/ana.21603 19399874 PMC2910719

[B40] Stafstrom C. E. Rho J. M. (2012). The ketogenic diet as a treatment paradigm for diverse neurological disorders. Front. Pharmacol. 3:59. 10.3389/fphar.2012.00059 22509165 PMC3321471

[B42] Straub H. Kohling R. Frieler A. Grigat M. Speckmann E. J. (2000). Contribution of l-type calcium channels to epileptiform activity in hippocampal and neocortical slices of guinea-pigs. Neuroscience 95, 63–72. 10.1016/s0306-4522(99)00401-7 10619462

[B43] Swann J. W. Brady R. J. (1984). Penicillin-induced epileptogenesis in immature rat CA3 hippocampal pyramidal cells. Brain Res. 314, 243–254. 10.1016/0165-3806(84)90046-4 6704751

[B44] Thakur K. T. Probasco J. C. Hocker S. E. Roehl K. Henry B. Kossoff E. H.. (2014). Ketogenic diet for adults in super-refractory status epilepticus. Neurology 82, 665–670. 10.1212/WNL.0000000000000151 24453083 PMC3945660

[B45] Thompson S. M. Wong R. K. (1991). Development of calcium current subtypes in isolated rat hippocampal pyramidal cells. J. Physiol. 439, 671–689. 10.1113/jphysiol.1991.sp018687 1654419 PMC1180129

[B46] Toyoda I. Fujita S. Thamattoor A. K. Buckmaster P. S. (2015). Unit activity of hippocampal interneurons before spontaneous seizures in an animal model of temporal lobe epilepsy. J. Neurosci. 35, 6600–6618. 10.1523/JNEUROSCI.4786-14.2015 25904809 PMC4405565

[B47] Traub R. D. Wong R. K. (1983). Synchronized burst discharge in disinhibited hippocampal slice. II. Model of cellular mechanism. J. Neurophysiol. 49, 459–471. 10.1152/jn.1983.49.2.459 6300344

[B48] Turner T. J. Adams M. E. Dunlap K. (1992). Calcium channels coupled to glutamate release identified by omega-aga-iva. Science 258, 310–313. 10.1126/science.1357749 1357749

[B50] Vexler Z. S. Berrios M. Ursell P. C. Sola A. Ferriero D. M. Gregory G. A. (1999). Toxicity of fructose-1,6-bisphosphate in developing normoxic rats. Pharmacol. Toxicol. 84, 115–121. 10.1111/j.1600-0773.1999.tb00885.x 10193671

[B49] Vexler Z. S. Wong A. Francisco C. Manabat C. Christen S. Täuber M.. (2003). Fructose-1,6-bisphosphate preserves intracellular glutathione and protects cortical neurons against oxidative stress. Brain Res. 960, 90–98. 10.1016/s0006-8993(02)03777-0 12505661

[B51] Wang X. Hooks B. M. Sun Q. Q. (2014). Thorough gabaergic innervation of the entire axon initial segment revealed by an optogenetic ‘laserspritzer’. J. Physiol. 592, 4257–4276. 10.1113/jphysiol.2014.275719 25085892 PMC4215776

[B52] Wheeler T. J. Chien S. (2012). Characterization of the high-affinity uptake of fructose-1,6-bisphosphate by cardiac myocytes. Mol. Cell. Biochem. 366, 31–39. 10.1007/s11010-012-1279-x 22426779

[B53] Wheeler T. J. McCurdy J. M. denDekker A. Chien S. (2004). Permeability of fructose-1,6-bisphosphate in liposomes and cardiac myocytes. Mol. Cell. Biochem. 259, 105–114. 10.1023/b:mcbi.0000021356.89867.0d 15124914

[B54] Wong R. K. Prince D. A. (1978). Participation of calcium spikes during intrinsic burst firing in hippocampal neurons. Brain Res. 159, 385–390. 10.1016/0006-8993(78)90544-9 728808

[B55] Xu K. Stringer J. L. (2008). Antioxidants and free radical scavengers do not consistently delay seizure onset in animal models of acute seizures. Epilepsy Behav. 13, 77–82. 10.1016/j.yebeh.2008.03.002 18396108 PMC2486491

[B56] Yamakage M. Namiki A. (2002). Calcium channels—basic aspects of their structure, function and gene encoding; anesthetic action on the channels—a review. Can. J. Anaesth. 49, 151–164. 10.1007/bf03020488 11823393

[B57] Yekhlef L. Breschi G. L. Lagostena L. Russo G. Taverna S. (2015). Selective activation of parvalbumin- or somatostatin-expressing interneurons triggers epileptic seizurelike activity in mouse medial entorhinal cortex. J. Neurophysiol. 113, 1616–1630. 10.1152/jn.00841.2014 25505119

